# Clinical evaluation of peri-implantitis relation with implant material

**DOI:** 10.6026/973206300223086

**Published:** 2026-05-31

**Authors:** Mokshada Badadare, Mamta Singh, Miftah Ur Rahman Md, Megha Modi, Nitin Gorwade, Anukrity Chandra

**Affiliations:** 1 Department of Prosthodontics, Bharati Vidyapeeth Dental College and Hospital, Sangli, Maharashtra, India; 2 Department of Oral and Maxillofacial Surgery, Index Institute of Dental Sciences and Hospital, Indore, Madhya Pradesh, India; 3 Department of Prosthodontics, Crown and Bridge, SB Patil Institute for Dental Sciences and Research, Bidar, Karnataka, India; 4 Department of Oral and Maxillofacial Surgery, Advanced Clinical Fellowship Program, New York University College of Dentistry, New York, United States; 5 Department of Prosthodontics, Crown and Bridge, Rungta Dental College, Bhilai, Chhattisgarh, India

**Keywords:** Dental implant material, peri-implantitis, biocompatibility

## Abstract

Peri-implantitis, characterized by inflammation of peri-implant tissues and progressive bone loss, remains a major biological complication affecting long-term implant success. Material composition, surface characteristics and biocompatibility influence microbial colonization, host immune response and implant durability. Therefore, it is of interest to investigate the relationship between different dental implant materials and the incidence of peri-implantitis among patients treated at a tertiary dental center. Clinical parameters including probing depth, bleeding on probing, radiographic bone loss, plaque index and type of implant material were recorded and analyzed. Data shows that surface roughness and alloy composition influence peri-implant tissue response, with implants exhibiting rougher surfaces and greater plaque accumulation showing higher inflammation levels. Thus, we show that appropriate material selection, optimized surface engineering and meticulous maintenance are essential strategies to minimize peri-implantitis risk and ensure long-term implant stability.

## Background:

Due to its excellent success rates, functional stability and capacity to retain alveolar bone, dental implants have become the primary treatment option for replacing lost teeth. Osseointegration and long-term results have been further bettered by developments in surface engineering, surgical techniques and implant design. However, biological complications such as peri-implant mucositis and peri-implantitis remain major concerns affecting implant longevity [1, 2]. Peri-implantitis is defined as an inflammatory condition characterized by bleeding on probing, increased probing depth, suppuration and progressive peri-implant bone loss following successful osseointegration [3]. Its prevalence has been reported to range from 10-25% of implants and up to 40% of patients, making it one of the most significant causes of late implant failure [4, 5]. The etiology of peri-implantitis is multifactorial and involves bacterial biofilm formation, host immune response, systemic risk factors and implant-related characteristics. Among the many elements affecting peri-implant tissue response, dental implants' material makeup and surface characteristics are among the most important. Because of their superior mechanical qualities, high resistance to corrosion and exceptional biocompatibility, titanium and titanium alloys are the materials most often utilized for implanting [6]. Surface alterations such as sandblasting, acid-etching, plasma spraying, or anodization may improve osseointegration by creating a stable oxide layer of titanium. However, these methods can also affect bacterial adherence and plaque retention [7, 8]. Rough implant surfaces promote bone-to-implant contact, but they may also make implants more susceptible to microbial colonization and peri-implant inflammation, according to studies [9].

Osseointegration is better and more complex biofilms are harbored by implants with somewhat rough surfaces compared to those with smoother surfaces, according to experimental findings [10]. Therefore, surface properties reflect a fine line between encouraging bone repair and reducing the likelihood of infection. Zirconia and other alternative implant materials have been getting a lot of interest lately because of their possible reduced bacterial affinity and cosmetic benefits. Some clinical trials have shown that, in comparison to titanium, zirconia implants had a better soft tissue response and less plaque buildup [11, 12]. Nevertheless, there is a lack of long-term clinical data comparing the risk of peri-implantitis with zirconia and titanium implants and reports of their mechanical reliability and fracture resistance are contradictory [13]. Some of the elements that may contribute to inflammation around implants include the materials used, the rate of corrosion, the release of metal ions and the degree of surface deterioration. It is possible that inflammatory responses, osteolysis and poor bone repair might be triggered by the release of titanium particles into peri-implant tissues [14]. According to these results, the characteristics of implant materials may affect tissue stability, host immunological regulation and microbial colonization. Although the major variables that determine peri-implant disease are patient-related, such as smoking, diabetes and periodontitis history, implants material and surface features might influence the severity and course of the illness [15]. Therefore, it is of interest to investigate the relationship between different dental implant materials and the incidence of peri-implantitis among patients treated at a tertiary dental center.

## Materials and Methodology:

## Study design and setting:

The periodontology and oral implantology department of a tertiary dental care center was the site of this clinical observational investigation. Dental implant material and peri-implant tissue health were the subjects of this cross-sectional investigation.

## Study population:

Dental implant recipients who showed up for scheduled maintenance or follow-up appointments during the research period were eligible for screening. All implants had to be put at least 12 months before the test to provide the peri-implant tissue enough time to mature and for any diseases to be detected.

## Sample selection:

## Inclusion criteria:

[1] Patients aged ≥18 years

[2] Presence of at least one functional dental implant

[3] Implant loading completed for at least one year

[4] Availability of treatment records indicating implant material and surface type

## Exclusion criteria:

[1] Patients with systemic conditions affecting bone metabolism (except controlled diabetes)

[2] History of radiotherapy to head and neck region

[3] Implants placed less than one year prior

[4] Incomplete clinical or radiographic records 

## Grouping based on implant material:

Implants were categorized according to their material composition:

[1] Group I: Commercially pure titanium implants

[2] Group II: Titanium alloy implants

[3] Group III: Zirconia implants (where applicable)

## Clinical examination:

A standardized clinical protocol was followed. All measurements were recorded by a calibrated examiner to minimize inter-observer variability.

## The following peri-implant parameters were assessed:

[1] Probing Depth (PD): measured at six sites per implant using a periodontal probe

[2] Bleeding on Probing (BOP): recorded as present or absent

[3] Plaque Index (PI): assessed around implant surfaces

[4] Suppuration: noted where present

[5] Clinical attachment level where measurable

## Radiographic assessment:

The paralleling approach was used to acquire standardized intraoral periapical radiographs. From the implant shoulder until the first bone-implant contact on both the mesial and distal sides, the measurement of marginal bone loss was taken. Analyzed were the mean values of bone loss.

## Definition of peri-implantitis:

Peri-implantitis was diagnosed based on the presence of:

[1] Probing depth ≥5 mm

[2] Bleeding and/or suppuration on probing

[3] Radiographic bone loss ≥2 mm beyond initial remodeling

## Data collection:

The following pieces of demographic and risk factor data were collected: Age, sex, smoking status, behaviors regarding oral hygiene, systemic diseases and the site of the implant. From the patient's medical record, we were able to determine the implant's material, surface properties and loading time.

## Outcome measures:

The frequency of peri-implantitis among categories of implant materials was the major end result. Clinical metrics such bleeding ratings, probing depth and marginal bone loss were compared across materials as secondary results.

## Statistical analysis:

Data were entered into statistical software for analysis. Continuous variables were expressed as mean ± standard deviation. Categorical variables were expressed as percentages. A p-value <0.05 was considered statistically significant

## Results:

Radiographic and clinical evaluations were conducted on 180 implants in 132 individuals. Implants were classified into three groups according to their material: Zirconia, titanium alloy and titanium. To establish a connection between implant material and peri-implantitis, we examined clinical peri-implant parameters in addition to bone levels. Zirconia made up 16.6% of the implants, titanium alloy 26.7% and commercially pure titanium 56.7% (Table 1). The distribution of these results shows how common titanium implants are in clinical settings. There was no discernible variation in demographics across the groups (p = 0.42), suggesting that they were comparable. Peri-implantitis was more common in certain materials than others. With a frequency of 31.2%, titanium alloy implants were the most common, followed by titanium (21.6%) and finally zirconia (13.3%). It seems that material composition may impact the probability of peri-implant inflammation, as there was a statistically significant difference between the groups (p = 0.04) (Table 2). The mean probing depth was highest around titanium alloy implants (4.5 ± 1.3 mm), followed by titanium implants (3.9 ± 1.2 mm), and lowest around zirconia implants (3.4 ± 1.1 mm). Similarly, bleeding on probing was greatest in the titanium alloy group (39.6%), compared to titanium (28.4%) and zirconia implants (19.3%). Plaque index values followed the same trend, with titanium alloy implants showing the highest plaque accumulation (40.8%), followed by titanium (34.1%), and zirconia implants demonstrating the lowest levels (24.6%). These differences were statistically significant across all parameters (p < 0.05), indicating that implant material composition and surface characteristics may influence peri-implant tissue health (Table 3). With 37.5% showing ≥2 mm bone resorption, titanium alloy implants had the highest marginal bone loss compared to titanium implants at 25.5% and zirconia implants at 16.7%. This disparity was statistically significant (p = 0.03), indicating that the material of the implant might affect the stability of the bone around the implant (Table 4).

## Discussion:

Progressive bone loss surrounding dental implants is defined by peri-implantitis, an inflammatory illness with several causes. Although microbial biofilm is still the major cause, new research highlights how biomechanical qualities, surface features and implant material affect the reaction of peri-implant tissues and the likelihood of infection. Because of its exceptional osseointegration, mechanical strength and longevity, titanium implants are still considered the best option in implant dentistry. Titanium implants, according to systematic analyses, have better stability of peri-implant tissues around titanium fixtures, which means they have a higher survival rate and less marginal bone loss than zirconia implants in short-term follow-up studies (77.6% vs 70.3%, p<0.05). But zirconia implants have been the talk of the town due to their less bacterial adherence and good cosmetic results. Based on clinical investigations, zirconia implants have a potential benefit in reducing peri-implant inflammation [16] because they develop less plaque and exhibit less microbial contamination than titanium implants. Under inflammatory challenge conditions, experimental models of mucositis showed that bleeding scores and plaque accumulation around titanium implants increased more than around zirconia implants. This lends credence to the idea that zirconia may have a lower inflammatory response in patients who are susceptible. In spite of these results, the two materials seem to have quite similar long-term biological effects. Under controlled oral hygiene circumstances, many comparison investigations have shown that peri-implant health measures such probing depth, bleeding on probing and plaque index are not significantly different [11, 17]. Titanium remains superior to zirconia in terms of biomechanics. Zirconia implants may be somewhat more prone to fractures or early failures because of their lower fracture toughness and structural stiffness, in contrast to titanium implants, which exhibit less marginal bone loss and better mechanical dependability. Zirconia implants may have a slightly greater peri-implantitis incidence (around 4% versus 2%), according to some recent clinical data; nevertheless, these differences are minor and usually not statistically significant [14]. While zirconia implants often show lower plaque accumulation and reduced microbial contamination compared with titanium, evidence for differences in bleeding on probing or marginal bone loss is inconsistent and confounded by study quality and patient-level factors. Therefore, implant material alone cannot be regarded as a definitive determinant of peri-implantitis risk [18]. All things considered, the results of the current clinical evaluation are in line with the existing body of research that suggests implant material affects peri-implant health, but is not the main factor that determines peri-implantitis. Implants made of zirconia may provide slight benefits in terms of aesthetics and plaque management, but titanium implants are demonstrably more structurally reliable.

## Conclusion:

There is a weak correlation between implant material and peri-implant tissue response, within the parameters of the research. Zirconia implants may show significantly less plaque buildup and inflammatory response, whereas titanium implants have better survival and mechanical stability. Although implant material does have a role in peri-implantitis, patient-related and prosthetic variables continue to have a greater impact. The most important factors that determine the success of implants are proper care, management of plaque and risk evaluation.

## Figures and Tables

**Table 1:** Distribution of implants according to material type

**Implant Material**	**Number of Implants**	**Percentage**
Commercially pure titanium	102	56.70%
Titanium alloy	48	26.70%
Zirconia	30	16.60%

**Table 2:** Prevalence of peri-implantitis by implant material

**Implant Material**	**Peri-Implantitis Present**	**Percentage**	**p-value**
Titanium	22 / 102	21.60%	-
Titanium alloy	15 / 48	31.20%	0.04
Zirconia	30-Apr	13.30%	0.04

**Table 3:** Comparison of clinical parameters around different implant materials

**Parameter**	**Titanium (Mean ± SD)**	**Titanium alloy (Mean ± SD)**	**Zirconia (Mean ± SD)**	**p-value**
Probing depth (mm)	3.9 ± 1.2	4.5 ± 1.3	3.4 ± 1.1	0.02
Bleeding on probing (%)	28.40%	39.60%	19.30%	0.01
Plaque index (%)	34.10%	40.80%	24.60%	0.03

**Table 4:** Marginal bone loss according to implant material

**Implant Material**	**Mean Bone Loss (mm)**	**Implants with Bone Loss≥2 mm**	**Percentage**	**p-value**
Titanium	1.62 ± 0.84	26	25.50%	-
Titanium alloy	2.01 ± 0.92	18	37.50%	0.03
Zirconia	1.31 ± 0.71	5	16.70%	0.03
